# Head-to-head performance comparison of self-collected nasal versus professional-collected nasopharyngeal swab for a WHO-listed SARS-CoV-2 antigen-detecting rapid diagnostic test

**DOI:** 10.1007/s00430-021-00710-9

**Published:** 2021-05-24

**Authors:** Julian A. F. Klein, Lisa J. Krüger, Frank Tobian, Mary Gaeddert, Federica Lainati, Paul Schnitzler, Andreas K. Lindner, Olga Nikolai, B. Knorr, A. Welker, Margaretha de Vos, Jilian A. Sacks, Camille Escadafal, Claudia M. Denkinger, for the Study Team

**Affiliations:** 1 Division of Clinical Tropical Medicine, Centre of Infectious Diseases, Heidelberg University Hospital, Im Neuenheimer Feld 324, 69120 Heidelberg, Germany; 2 Department of Virology, Centre of Infectious Diseases, Heidelberg University Hospital, Heidelberg, Germany; 3 Charité – Universitätsmedizin Berlin, corporate member of Freie Universität Berlin and Humboldt-Universität zu Berlin, Institute of Tropical Medicine and International Health, Berlin, Germany; 4 Local Health Authority of Heidelberg and Rhein-Neckar-Region, Heidelberg, Germany; 5 Foundation for Innovative New Diagnostics, Geneva, Switzerland; 6 German Centre for Infection Research (DZIF), 69120 Heidelberg, Germany

**Keywords:** SARS-CoV-2, COVID-19, Nasal sampling, Antigen-detecting rapid diagnostic test, Self-sampling, Head-to-head comparison

## Abstract

In 2020, the World Health Organization (WHO) recommended two SARS-CoV-2 lateral flow antigen-detecting rapid diagnostics tests (Ag-RDTs), both initially with nasopharyngeal (NP) sample collection. Independent head-to-head studies are necessary for SARS-CoV-2 Ag-RDT nasal sampling to demonstrate comparability of performance with nasopharyngeal (NP) sampling. We conducted a head-to-head comparison study of a supervised, self-collected nasal mid-turbinate (NMT) swab and a professional-collected NP swab, using the Panbio™ Ag-RDT (distributed by Abbott). We calculated positive and negative percent agreement between the sampling methods as well as sensitivity and specificity for both sampling techniques compared to the reference standard reverse transcription polymerase chain reaction (RT-PCR). A SARS-CoV-2 infection could be diagnosed by RT-PCR in 45 of 290 participants (15.5%). Comparing the NMT and NP sampling the positive percent agreement of the Ag-RDT was 88.1% (37/42 PCR positives detected; CI 75.0–94.8%). The negative percent agreement was 98.8% (245/248; CI 96.5–99.6%). The overall sensitivity of Panbio with NMT sampling was 84.4% (38/45; CI 71.2–92.3%) and 88.9% (40/45; CI 76.5–95.5%) with NP sampling. Specificity was 99.2% (243/245; CI 97.1–99.8%) for both, NP and NMT sampling. The sensitivity of the Panbio test in participants with high viral load (> 7 log_10_ SARS-CoV-2 RNA copies/mL) was 96.3% (CI 81.7–99.8%) for both, NMT and NP sampling. For the Panbio supervised NMT self-sampling yields comparable results to NP sampling. This suggests that nasal self-sampling could be used for to enable scaled-up population testing.

**Clinical Trial** DRKS00021220.

**Supplementary Information:**

The online version contains supplementary material available at 10.1007/s00430-021-00710-9.

## Introduction

The use of antigen-detecting rapid diagnostic tests (Ag-RDTs) for SARS-CoV-2 has increased within the last months and has an important role in pandemic management. However, broader use and scale-up is limited due to complex sampling methods. In 2020, the World Health Organization (WHO) recommended two lateral flow Ag-RDTs ((SD Biosensor, Inc. Gyeonggi-do, Korea, distributed by Roche, Germany, henceforth called Standard Q; and Abbott Panbio™ (Rapid Diagnostics, Jena, Germany; henceforth called Panbio))), both initially with nasopharyngeal (NP) sample collection [1, 2]. Since then, independent head-to-head studies demonstrated that nasal sampling (including self-sampling) assessed against NP sampling leads to comparable performance using the SARS-CoV-2 Ag-RDT SD STANDARD Q [3–5]. For Panbio, only one study to date assessed professional nasal mid-turbinate (NMT) sampling and showed 82.1% sensitivity and 99.1% specificity in comparison to reverse transcription polymerase chain reaction (RT-PCR). However, a head-to-head comparison with NP sampling has not been performed to date [6].

## Materials and methods

We conducted a manufacturer-independent prospective study directly comparing the diagnostic accuracy of Panbio performed with a supervised, self-collected NMT swab versus a professionally collected NP swab. For the two Ag-RDT sampling techniques positive and negative percent agreements (PPA, NPA) were calculated. Sensitivity and specificity were assessed and compared against the reference standard RT-PCR.

The ethical review committee at Heidelberg University Hospital approved the study protocol (registration number S-180/2020). Enrollment and testing took place in Heidelberg (Germany) between December 15th 2020 and January 19th 2021 in a SARS-CoV-2 drive-in testing center, led by the local health authority. We included adults with symptoms suggestive for a SARS-CoV-2 infection or a recent high-risk contact with a confirmed SARS-CoV-2 case.

After written informed consent, each participant was instructed to self-collect a NMT swab for the Ag-RDT under supervision using a non-flocked swab (Jiangsu Changfeng Medical Industry Co., Ltd., Jiangsu, China), provided by Abbott in the research use only Panbio kit for nasal swab testing. The instructions were verbal and picture-guided following the manufacturer’s instructions for use. In a second step, a health worker collected a NP swab (using IMPROSWAB®, Guangzhou Improve Medical Instruments Co., Ltd., Guangzhou, China), for RT-PCR testing in one nostril. Finally, a second NP swab for Ag-RDT testing was collected from the patient using a nylon-flocked specimen (NFS-SWAB Applicator™, Noble Bioscienes Inc., Gyeonggi-do, Korea), provided with the commercial Abbott (nasopharyngeal) test kit. The Panbio was conducted on-site by trained study personnel following the manufacturer’s instruction for use for each kit [7]. Two study staff read out the Ag-RDT results, each of them blinded to the interpretation of the other.

For RT-PCR testing the Tib Molbiol® (Berlin, Germany) assay was used. Ribonucleic acid (RNA) was isolated from samples using QIAGEN Kits (QIAGEN, Hilden, Germany) automated on the QIASymphony instrument, extracted RNA was used for SARS-CoV-2 RT-PCR reaction targeting E-gene and N-gene of SARS-CoV-2 according to manufacturer's instructions (TIB MOLBIOL, Berlin, Germany). RT-PCR was performed on a LightCycler 480 instrument (Roche, Mannheim, Germany). The analytical sensitivity in this assay for SARS-CoV-2 positive samples is approximately 1000 copies/ml. The E-Gene of SARS-CoV-2 was used for the cycle threshold (Ct)-value determination and the viral load calculations. A conversion of the Ct-values into viral-load (VL) was performed using RT-PCR with defined amounts of quantified SARS-CoV-2 in vitro transcripts [8].

Leftover samples of the NMT and NP swabs resuspended in Ag-RDT buffer were stored at − 20°C. Samples that were identified to be false-positive in comparison to RT-PCR on one or both Ag-RDTs were retested with RT-PCR from the remnant Ag-RDT buffer.

## Results

We screened a total of 369 eligible individuals of whom 292 (79.1%) gave written consent. After exclusion of two participants (one with invalid RT-PCR result and one with lost written informed consent), 290 participants were included in the analysis (study flow detailed in Additional file 1: Figure S1). Our study population had an average age of 42.7 years (standard deviation (SD) 14.6), 33.8% (98/290) had comorbidities and 52.4% (152/290) were female. In total, 45.9% (133/290) were symptomatic on the day of testing with a mean duration of symptoms of 3.8 days (SD 5.4). SARS-CoV-2 infection was detected by RT-PCR in 15.5% (45/290) of the study population (Table 1), with eight infections being among asymptomatic participants. One invalid Ag-RDT was registered on NP samples, which was valid upon repeat.

**Table 1:** Ag-RDT results with a supervised self-collected nasal mid-turbinate (NMT) swab and professional-collected nasopharyngeal (NP) swab in RT-PCR positive patients

Ag-RDT (NMT swab) Self-collected	Ag-RDT (NP swab) Prof.-collected	RT-PCR (NP swab) professionally-collected		Symptom duration (days)
		Ct-value^a^	Viral load (log_10_ SARS-CoV-2 RNA copies/ml)	
Positive	Positive	12.7	10.0	2
Positive	Positive	12.9	9.9	4
Positive	Positive	13.1	9.9	3
Positive	Positive	16.1	9.0	3
Positive	Positive	16.4	8.9	3
Positive	Positive	16.5	8.9	2
Positive	Positive	16.5	8.9	1
Positive	Positive	16.6	8.9	1
Positive	Positive	16.7	8.8	1
Positive	Positive	17.8	8.5	0
Positive	Positive	17.9	8.5	1
Positive	Positive	18.8	8.2	2
Positive	Positive	18.8	8.2	7
Positive	Negative	18.9	8.2	1
Positive	Positive	19.5	8.0	5
Positive	Positive	19.7	7.9	1
Positive	Positive	19.9	7.9	4
Positive	Positive	19.9	7.9	Asymptomatic^c^
Positive	Positive	20.1	7.8	Asymptomatic^c^
Positive	Positive	20.2	7.8	10
Positive	Positive	21.2	7.5	2
Positive	Positive	21.4	7.4	Asymptomatic^c^
Negative	Positive	22.1	7.2	1
Positive	Positive	22.5	7.1	5
Positive	Positive	22.5	7.1	2
Positive	Positive	22.6	7.1	6
Positive	Positive	22.8	7.0	5
Positive	Positive	23.1	6.9	Asymptomatic^c^
Positive	Positive	23.1	6.9	1
Positive	Positive	23.6	6.8	5
Positive	Positive	23.8	6.7	2
Positive	Positive	25.7	6.2	2
Positive	Positive	25.9	6.1	7
Positive	Positive	26.0	6.1	6
Positive	Positive	26.3	6.0	Asymptomatic^c^
Positive	Positive	26.7	5.9	1
Negative	Negative^d^	26.7	5.9	1
Positive	Positive	27.7	5.6	2
Negative	Positive	29.7	5.0	Asymptomatic^c^
Negative	Negative	30.6	4.7	Asymptomatic^c^
Positive	Positive	31.2	4.5	1
Positive	Negative	31.2	4.5	1
Negative	Positive	32.7	4.1	n.a
Negative	Positive	33.8	3.8	Asymptomatic^#^
Negative	Negative	34.5	3.6	10
Sensitivity 84.4% (38/45; CI 71.2–92.3%)	Sensitivity 88.9% (40/45; CI 76.5–95.5%)			
Positive Percent Agreement 88.1% (CI 75.0–94.8%)^b^				

*Ag-RDT* antigen-detecting rapid diagnostic test, *NMT* nasal mid-turbinate, *NP* nasopharyngeal, *Ct* cycle threshold, *RT-PCR* reverse transcription-polymerase chain reaction, *n.a.* not available, *CI* confidence interval

^a^Assay: TibMolBiol

^b^Including one false-positive on NMT and NP and one on NP

^c^On the day of testing

^d^Oropharyngeal swab due to contraindications of NP Sampling

The overall sensitivity of Panbio with NP sampling was 88.9% (40/45; 95% confidence interval (CI) 76.5–95.5%) and 84.4% (38/45; CI 71.2–92.3%) with NMT sampling. Four infections were identified by NP Ag-RDT sampling, which were negative in NMT sampling of which two had a low VL (VL < 4.9 log_10_ SARS-CoV-2 RNA copies/ml) and two were asymptomatic (Table 1). Two participants had a positive NMT result, not detected via NP Ag-RDT, of which one had a low VL (VL < 4.9 log_10_ SARS-CoV-2 RNA copies/ml). Specificity was 99.2% (243/245; CI 97.1–99.8%) for both, NP and NMT sampling. Considering only RT-PCR positive participants with high VL (> 7 log_10_ SARS-CoV-2 RNA copies/mL), the sensitivity of the Panbio test was 96.3% (CI 81.7–99.8%) for both NMT and NP sampling. Excluding nine participants with oropharyngeal sampling instead of NP (due to contraindications of NP sampling) increased sensitivity only marginally (40/44; 90.9% (CI 78.8–96.4%)). Detailed results by symptoms and sub-group analyses are available in Additional file 1: Tables S2, S3. The positive percent agreement of the Ag-RDT was 88.1% (37/42 PCR positives detected; CI 75.0–94.8%) including one false-positive by both NMT and NP, and one false-positive by NP only. The negative percent agreement was 98.8% (245/248; CI 96.5–99.6%). Inter-rater reliability for the interpretation of the Ag-RDTs was perfect with a kappa of 1.0. Participants reported NMT sampling to be better tolerated than NP sampling.

When performing RT-PCR from the remnant buffer/sample-mixture, SARS CoV-2 was identified in both NMT and NP samples from the same participant with a false-positive Ag-RDT result. This suggests the Ag-RDT result being in fact true-positive with a sampling error likely having occurred for the RT-PCR from NP sample. For two other false-positives, one each on NMT and NP, no virus was identified in the buffer solution. Among three false-negative NMT samples one buffer was positive with low VL (4.38 log_10_ SARS-CoV-2 RNA copies/ml; Additional file 1: Table S4, suggesting that the VL was below the limit of detection of the Ag-RDT.

## Discussion

Our study demonstrates that the overall sensitivity of Panbio with NP (88.9%) and NMT sampling (84.4%) is comparable. Both sampling techniques, NP and NMT sampling, yielded a specificity of 99.2%. Our findings for performance are similar to findings of other studies that evaluated the Panbio [6, 9, 10]. Also, the performance in our study is corroborated by an evaluation of analytical sensitivity and exclusivity in a study by Corman et al. showing the Ag-RDT to have excellent performance with the viral load as observed in the first week of disease [11]. Berger et al. found the Panbio to also perform similar to the Roche SARS-CoV-2 Ag-RDT (which is also distributed in parts of the world by the original equipment manufacturer SD Biosensor as STANDARD™ Q COVID-19 Ag) [10]. And the two tests were confirmed to be the best performing Ag-RDTs in two meta-analyses recently published on the topic [12, 13]. Studies that show lower sensitivity on either of the two tests (e.g. 64.5% by Osterman et al. [14] for Roche SARS-CoV-2 Ag-RDT or Olearo et al. [15] with 44.6% for Panbio in symptomatic patients), most commonly perform the test not according to manufacturer’s recommendations (most often either not using the sample type or swab recommended, or prediluting the sample in transport media). This possible reason for the difference in performance between studies was also highlighted in the recent systematic reviews.

The percentage positive in our study was 15.5%, similar to the overall percentage positive in Germany at the time of the study. While this is much higher than expected from the incidence in the region, the high percentage positive reflects the national test strategy which results in a preselection of tested individuals done by the local health authority [16].

Our study has several strengths. Study methods were rigorous and included standardized sampling and two independent blinded readers. The study population is representative, judging from the similar sensitivity of the Panbio test with NP sampling observed in our study in comparison to two large validation studies [9, 10]. All samples for routine RT-PCR were tested via the same RT-PCR assay (Table 1). The RT-PCR on the leftover buffer solution of Ag-RDT allowed us to perform further discrepant analysis.

A limitation of the study is that it was performed in a single center. The preselection of participants invited to come for testing was done according to national guidelines. We did not record deviations from the recommended NMT procedure, however, as the sampling was done under proactive supervision, no major deviations were observed. Readers were not blinded to the sampling method while interpreting the test results, but weak positive results are rarely observed with the Panbio test, thus this limitation is unlikely to result in a difference in result interpretation. In the discrepant analysis, we did not perform RT-PCR of all Ag-RDT buffer solutions thus introducing a possible bias.

Our study suggests that supervised NMT self-sampling leads to results comparable to NP sampling for the Panbio Ag-RDT. A possible reduction in VL present in the nasal region compared to the nasopharyngeal region may be counterbalanced by the ease-of-sampling. Results of nasal sampling could potentially be further improved, if flocked swabs were used [17]. Standardized easy self-sampling methods are highly desirable, as they could increase throughput and require fewer medical personnel, which is often a bottle neck for scaling of antigen testing.

## Supplementary Information

Below is the link to the electronic supplementary material.

Additional file 1. Table S1: Study Team. Figure S1: Study Flow. Table S2: Detailed list of symptoms for all PCR positive participants. Table S3: Sensitivity and Specificity overall and by subgroups. Table S4: Ag-RDT – RT-PCR discrepant analysis: Buffer solution RT-PCR-results of Ag-RDT false-positive and Ag-RDT false-negative retained samples.

## Data Availability

All data generated or analyzed during this study are included in this published article and its Additional file.
