# Supplementary material for: Head-to-head performance comparison of self-collected nasal versus professional-collected nasopharyngeal swab for a WHO-listed SARS-CoV-2 antigen-detecting rapid diagnostic test
Source: Med Microbiol Immunol. 2021 May 24;210(4):181–6. doi: 10.1007/s00430-021-00710-9 (PMC8142294; doi:10.1007/s00430-021-00710-9)
Supplement: Supplementary file 1 — Additional file 1. Table S1: Study Team. Figure S1: Study Flow. Table S2: Detailed list of symptoms for all PCR positive participants. Table S3: Sensitivity and Specificity overall and by subgroups. Table S4: Ag-RDT – RT-PCR discrepant analysis: Buffer solution RT-PCR-results of Ag-RDT false-positive and Ag-RDT false-negative retained samples. [file 430_2021_710_MOESM1_ESM.docx]

**Head-to-head performance comparison** **of self-collected nasal** *versus* **professional-collected nasopharyngeal swab for a WHO-listed SARS-CoV-2 antigen-detecting rapid diagnostic test**

**Additional Material**

**Julian A.F. Klein^1^, Lisa J. Krüger^1^, Frank Tobian^1^, Mary Gaeddert^1^, Federica Lainati^1^, Paul Schnitzler^2^, Andreas K. Lindner^3^, Olga Nikolai^3^, B. Knorr^4^, A. Welker^4^, Margaretha de Vos^5^, Jilian A. Sacks^5^, Camille Escadafal^5^, Claudia M. Denkinger^1,6§^ for the study team**

^1^Division of Clinical Tropical Medicine, Centre of Infectious Diseases, Heidelberg University Hospital, Germany

^2^Department of Virology, Centre of Infectious Diseases, Heidelberg University Hospital, Germany

^3^Charité – Universitätsmedizin Berlin, corporate member of Freie Universität Berlin, Humboldt-Universität zu Berlin, and Berlin Institute of Health, Institute of Tropical Medicine and International Health, Berlin, Germany

^4^Local Health Authority of Heidelberg and Rhein-Neckar-Region, Germany

^5^Foundation for Innovative New Diagnostics, Geneva, Switzerland

^6^German Centre for Infection Research (DZIF), partner site Heidelberg, 69120 Heidelberg, Germany

**Correspondence:** Claudia M. Denkinger, Division of Clinical Tropical Medicine, Heidelberg University Hospital, Im Neuenheimer Feld 324, 69120 Heidelberg, Germany. E-mail: [claudia.denkinger@uni-heidelberg.de](mailto:claudia.denkinger@uni-heidelberg.de)

**Table of content**

[**(A)** **Table S1: Study Team** 3](#_Toc72476619)

[**(B)** **Figure S1: Study Flow** 4](#_Toc72476620)

[**(C)** **Table S2: Detailed list of symptoms for all PCR positive participants** 5](#_Toc72476621)

[**(D)** **Table S3: Sensitivity and Specificity overall and by subgroups** 7](#_Toc72476622)

[**(E)** **Table S4: Ag-RDT – RT-PCR discrepant analysis: Buffer solution RT-PCR-results of Ag-RDT false-positive and Ag-RDT false-negative retained samples** 8](#_Toc72476623)

# **Table S1: Study Team**

| Public Health Authority, Rhein-Neckar-Region, Heidelberg, Germany | Dr. K. Assaad, |
| --- | --- |
|  | Dr. A. Fuhs |
|  | Dr. C. Harter |
|  | C. Schulze |
|  | G. Schmitt |
| Division of Clinical Tropical Medicine, Heidelberg University Hospital, Heidelberg Germany | Loai Abutaima |
|  | Rico Müller |
|  | Martina Fink |
|  | Mathilde Fougereau |
|  | Maximilian Schirmer |
|  | Annika Small |
|  | Matthias Meinlschmidt |
|  | Valerie Dürr |
|  | Alina Schuckert |
|  | Ann-Kathrin Backes |
|  | Salome Steinke |
|  | Henrik Ellinghaus |
|  | Magdalena Mikula |
|  | Nele Schäfer |

# **Figure S1: Study Flow**


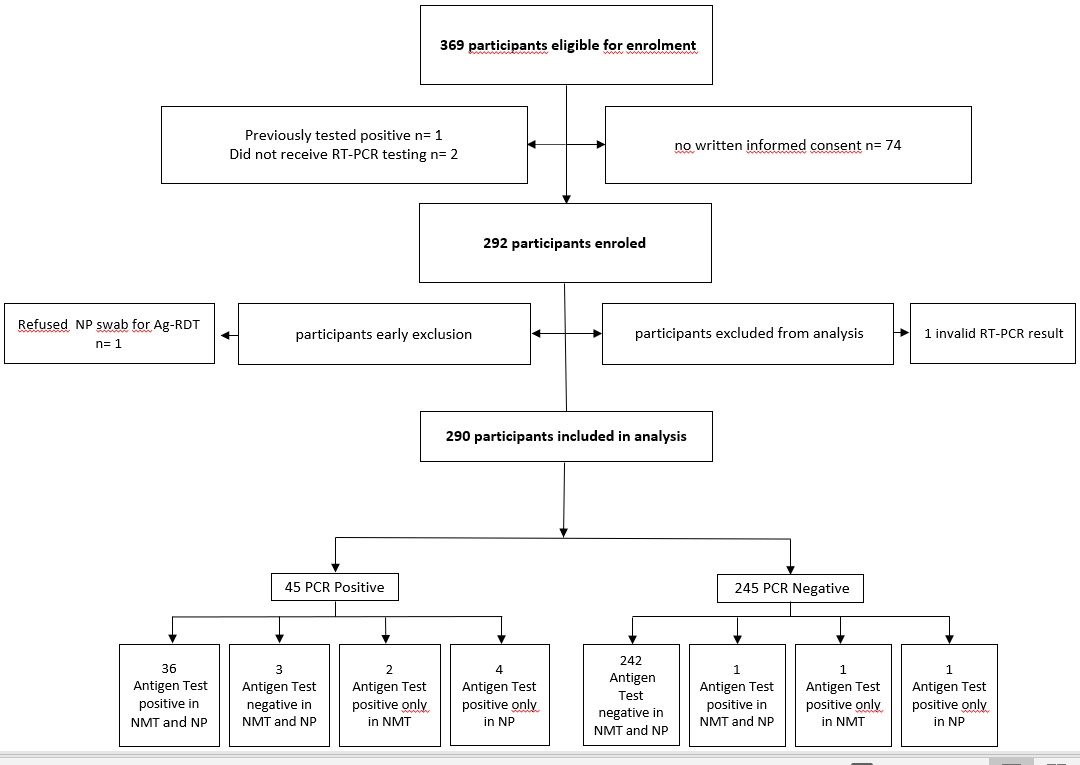


#

1. **Table S2: Detailed list of symptoms for all PCR positive participants**

| **CT value:**  **E-Gene** | **Viral load**  (log_10_RNA copies/mL) | **Result Ag-RDT NMT swab** | **Result Ag-RDT NP swab** | **Increased temperature/fever** | **Cough** | **Productive Cough** | **Sore throat** | **Dyspnoe** | **Body aches/**  **muscle pain** | **Fatigue** | **Headache** | **Runny**  **nose** | **Chest pain** | **Diarrhea** | **Nausea / Vomitus** | **Loss of taste/ smell** | **Other symptoms** |
| --- | --- | --- | --- | --- | --- | --- | --- | --- | --- | --- | --- | --- | --- | --- | --- | --- | --- |
| 13.1 | 9.9 | positive | positive | No | Yes | No | No | No | Yes | Yes | Yes | No | No | No | No | No | Yes |
| 25.7 | 6.2 | positive | positive | No | Yes | No | No | No | Yes | Yes | Yes | No | No | Yes | No | No | No |
| 22.6 | 7.1 | positive | positive | Yes | Yes | No | Yes | No | Yes | Yes | Yes | No | Yes | No | No | Yes | No |
| 31.2 | 4.5 | positive | negative | No | No | No | Yes | No | Yes | Yes | Yes | No | No | No | No | No | No |
| 26.0 | 6.1 | positive | positive | No | No | No | No | No | No | No | Yes | Yes | No | No | No | Yes | No |
| 22.1 | 7.2 | negative | positive | Yes | Yes | No | Yes | No | Yes | Yes | Yes | No | No | Yes | Yes | No |  |
| 23.1 | 6.9 | positive | positive |  |  |  |  |  |  |  |  |  |  |  |  |  |  |
| 26.7 | 5.9 | positive | positive | No | No | No | No | No | No | No | No | No | No | No | No | Yes | No |
| 16.1 | 9.0 | positive | positive | No | Yes | Yes | Yes | No | Yes | Yes | No | Yes | No | No | No | No | No |
| 16.5 | 8.9 | positive | positive | Yes | Yes | No | Yes |  | No | Yes | Yes | No | No | No | No | No | No |
| 19.7 | 7.9 | positive | positive | No | No | No | Yes | No | No | Yes | Yes | No | No | No | No | No | No |
| 26.7 | 5.9 | negative | negative | No | No | No | No | No | No | No | Yes | No | No | No | No | No | No |
| 16.6 | 8.9 | positive | positive | Yes | No | No | No | No | No | No | Yes | No | No | No | No |  | No |
| 18.9 | 8.2 | positive | negative | No | No | No | No | No | No | No | No | Yes | No | No | No | No | Yes |
| 23.8 | 6.7 | positive | positive | No | No | No | No | No | Yes | Yes | No | Yes |  | No | No | Yes | No |
| 16.7 | 8.8 | positive | positive | No | Yes | No | Yes | No | Yes | Yes | Yes | No | Yes | No | No | No | No |
| 17.8 | 8.5 | positive | positive | Yes | No | No | No | No | Yes | No | No | No | No | No | No | No | No |
| 20.1 | 7.8 | positive | positive |  |  |  |  |  |  |  |  |  |  |  |  |  |  |
| 16.4 | 8.9 | positive | positive | No | Yes |  | Yes | No | No | Yes | Yes | No | No | No | No | No | No |
| 30.6 | 4.7 | negative | negative |  |  |  |  |  |  |  |  |  |  |  |  |  |  |
| 17.9 | 8.5 | positive | positive | Yes | No | No | No | No | Yes | Yes | Yes | No | No | No | No | No | No |
| 21.2 | 7.5 | positive | positive | Yes | No | Yes | Yes | No | Yes | Yes | Yes | No | Yes | No | No | No | Yes |
| 18.8 | 8.2 | positive | positive | Yes | No | No | Yes | No | Yes | Yes | Yes | Yes | No | No | No | No | No |
| 27.7 | 5.6 | positive | positive | Yes | Yes | Yes | No | No | Yes | Yes | Yes | No | No | No | No | Yes | No |
| 12.7 | 10.0 | positive | positive | No | Yes | Yes | No | No | No | Yes | Yes | Yes | No | No | No | No | No |
| 16.5 | 8.9 | positive | positive | No | Yes | Yes | No | No | Yes | No | Yes | Yes | No | Yes | No | No | No |
| 33.8 | 3.8 | negative | positive |  |  |  |  |  |  |  |  |  |  |  |  |  |  |
| 22.5 | 7.1 | positive | positive | No | No | No | No | No | Yes | No | Yes | No | No | No | No | Yes | No |
| 12.9 | 9.9 | positive | positive | Yes | Yes | Yes | Yes | No | No | Yes | No | Yes | No | No | No | No | No |
| 25.9 | 6.1 | positive | positive | Yes | Yes | Yes | No | No | Yes | Yes | Yes | Yes | No | No | No | No | No |
| 18.8 | 8.2 | positive | positive | Yes | No | No | No | No | Yes | Yes | Yes | No | No | No | No | Yes | Yes |
| 23.6 | 6.8 | positive | positive |  | Yes | No | Yes | No | Yes | Yes | Yes | Yes | No | No | No | Yes |  |
| 34.5 | 3.6 | negative | negative | No | Yes | No | No | No | No | No | No | Yes | No | No | No | No | No |
| 19.9 | 7.9 | positive | positive | No | Yes | No | Yes | No | No | Yes | Yes | No | No | Yes | No | Yes | No |
| 23.1 | 6.9 | positive | positive | No | Yes | Yes | Yes | No | Yes | Yes | Yes | Yes | No | No | Yes | No | No |
| 31.2 | 4.5 | positive | positive | No | No | No | No | No | No | Yes | Yes | Yes | No | Yes | No | Yes | No |
| 21.4 | 7.4 | positive | positive |  |  |  |  |  |  |  |  |  |  |  |  |  |  |
| 29.7 | 5.0 | negative | positive |  |  |  |  |  |  |  |  |  |  |  |  |  |  |
| 22.8 | 7.0 | positive | positive | No | Yes | No | No | No | Yes | Yes | Yes | No | Yes | No | No | Yes | No |
| 22.5 | 7.1 | positive | positive | Yes | Yes | Yes | No | Yes | No | Yes | Yes | No | No | No | No | No | No |
| 19.9 | 7.9 | positive | positive |  |  |  |  |  |  |  |  |  |  |  |  |  |  |
| 26.3 | 6.0 | positive | positive |  |  |  |  |  |  |  |  |  |  |  |  |  |  |
| 32.7 | 4.1 | negative | positive | Yes | Yes | No | Yes | Yes | Yes | Yes | Yes | Yes | Yes | No | No | Yes | No |
| 20.2 | 7.8 | positive | positive | Yes | Yes | No | No | No | Yes | Yes | Yes | Yes | No | No | No | Yes | No |
| 19.5 | 8.0 | positive | positive | No | Yes | No | No | No | Yes | No | Yes | Yes | No | No | No | No | No |

# **Table S3: Sensitivity and Specificity overall and by subgroups**

|  | **Sampling technique** | **Sensitivity** (%; 95% CI) | **Specificity** (%; 95% CI) | **Positive Percent Agreement** | **Negative Percent Agreement** |
| --- | --- | --- | --- | --- | --- |
| **Overall** | NP | 40* / 45 (88.9%; 76.5% - 95.5%) | 243 / 245 (99.2%; 97.1% - 99.8%) | 37* / 42** (88.1%; 75.0% - 94.8%) | 245 / 248 (98.8%; 96.5% - 99.6%) |
|  | NMT | 38 / 45 (84.4%; 71.2% - 92.3%) | 243 / 245 (99.2%; 97.1% - 99.8%) |  |  |
| **Viral load**  **≥7 log10**  *SARS-CoV-2 RNA copies/ml* | NP | 26 / 27 (96.3%; 81.7% - 99.8%) | n.a. | 25 / 26 (96.2%; 81.1% - 99.8%) | n.a. |
|  | NMT | 26 / 27 (96.3%; 81.7% - 99.8%) | n.a. |  |  |
| **Viral load**  **<7 log10**  *SARS-CoV-2 RNA copies/ml* | NP | 14 / 18 (77.8%; 54.8% - 90.1%) | n.a. | 11 / 14 (78.6%; 52.4% - 92.4%) | n.a. |
|  | NMT | 12 / 18 (66.7%; 43.7% - 83.7%) | n.a. |  |  |
| **symptomatic** | NP | 33/37 (89.2%, 75.3% - 95.7%) | 94/96 (97.9%, 81.4% - 99.4%) | 33 / 35 (91.4%, 77.6% - 97.0%) | 96 / 98 (98.0%, 92.8% - 99.4%) |
|  | NMT | 33/37 (89.2%, 75.3% - 95.7%) | 95/96 (99.0%, 94.3% - 99.9%) |  |  |
| **asymptomatic** | NP | 7/8 (87.5%, 52.9% - 99.4%) | 147/147 (100%, 97.5 - 100%) | 5 / 7 (71.4%, 35.9% - 91.8%) | 147 / 148 (99.3%, 96.3% - 100%) |
|  | NMT | 5/8 (62.5%, 30.6% - 86.3%) | 146/147 (99.3%, 96.2% - 100%) |  |  |

Abbreviations: n.a.: not applicable; NP: nasopharyngeal; NMT: nasal mid-turbinate; CI: confidence interval

*including one false-positive in NMT and NP

**including two false-positive in NP

# **Table S4: Ag-RDT – RT-PCR discrepant analysis: Buffer solution RT-PCR-results of Ag-RDT false-positive and Ag-RDT false-negative retained samples**

| **Ag-RDT false-positives** | | | | | | | | |
| --- | --- | --- | --- | --- | --- | --- | --- | --- |
| **Ag-RDT NMT swab** | **Ag-RDT NP swab** | **RT-PCR** | **Assay** | **RT-PCR buffer NMT sample** | **RT-PCR buffer NP sample** | **Sampling**  **comment** | **Probe**  **aspect** | **Symptom duration**  **(days)** |
| positive | negative | negative | TibMolBiol | negative | negative | none | NMT: mucous NP: clear | asymptomatic |
| positive | positive | negative | TibMolBiol | positive  (Ct 27.3; VL* 6.3) | positive  (Ct 31.8; VL* 5.0 | none | NMT: clear NP: mucous | 3 |
| negative | positive | negative | TibMolBiol | negative | negative | none | NMT: clear NP: mucous | 6 |

| **Ag-RDT false-negatives** | | | | | | | | | |
| --- | --- | --- | --- | --- | --- | --- | --- | --- | --- |
| **Ag-RDT NMT swab** | **Ag-RDT NP swab** | **Ct-value** | **Viral load*** | **Assay** | **RT-PCR buffer NMT sample** | **RT-PCR buffer NP sample** | **Sampling**  **comment** | **Probe**  **aspect** | **Symptom duration (days)** |
| negative | positive | 22.1 | 7.2 | TibMolBiol | sample got lost | not tested | none | NMT: clear NP:clear | 1 |
| negative | positive | 29.7 | 5.0 | TibMolBiol | negative | not tested | none | NMT: mucous NP:clear | asymptomatic |
| negative | positive | 32.7 | 4.1 | TibMolBiol | positive  (Ct 33.7; VL* 4.4) | not tested | none | NMT: mucous  NP: mucous,bloody spots | n.a. |
| negative | positive | 33.8 | 3.8 | TibMolBiol | negative | not tested | none | NMT: clear  NP: mucous | asymptomatic |
| positive | negative | 18.9 | 8.2 | TibMolBiol | not tested | negative | none | NMT: clear NP:clear | 1 |
| positive | negative | 31.2 | 4.5 | TibMolBiol | not tested | sample got lost | none | NMT: clear NP:clear | 1 |
| Ct: cycle threshold; Ag-RDT: antigen-detecting rapid diagnostic test; NMT: nasal mid-turbinate; NP: nasopharyngeal. n.a.: not available; *log_10_ *SARS-CoV-2* RNA copies/ml | | | | | | | | | |
